# La lutte contre le COVID-19 au Niger: l´évaluation de la prévention et contrôle des infections dans les formations sanitaires de la communauté urbaine de Niamey

**DOI:** 10.11604/pamj.supp.2020.37.1.26512

**Published:** 2020-11-11

**Authors:** Abdoulaye Mariama Baissa, Samba Hamani, Mahamane Ali, Arlette Leufak Mouako, Blanche-Philomene Melanga Anya, Charles Shey Wiysonge

**Affiliations:** 1 Organisation Mondiale de la Santé, Bureau Pays, Quartier Plateau, Avenue Mohamed VI 1204, Niamey, Niger,; 2 Ministère de la Santé Publique, Place des Ministères, BP 623, Niamey, Niger,; 3 Department of Global Health, Stellenbosch University, Francie van Zijl Drive, Tygerberg 7505, Cape Town, South Africa,; 4 Cochrane South Africa, South African Medical Research Council, Francie van Zijl Drive, Parow Valley 7501, Cape Town, South Africa,; 5 School of Public Health and Family Medicine, University of Cape Town, Anzio Road, Observatory 7935, Cape Town, South Africa

**Keywords:** Prévention et contrôle des infections, formation sanitaire, Niamey, Niger, Infection prevention and control, healthcare facility, Niamey, Niger

## Abstract

Le Niger fait face depuis le 19 mars 2020, à la pandémie de maladie à coronavirus 2019 (COVID-19). L´objectif de cette étude était d´évaluer les mesures de prévention et de contrôle des infections (PCI) dans les structures sanitaires de la communauté urbaine de Niamey au Niger et d´élaborer un plan de résolution des problèmes. Il s´agissait d´une analyse descriptive des 12 thématiques contenues dans la fiche Scorecard PCI de l´Organisation Mondiale de la Santé au niveau des formations sanitaires (FS) publiques et privées de la communauté urbaine de Niamey. L´étude s´est basée sur 83 formations sanitaires, soit 60% des formations sanitaires de la communauté urbaine de Niamey. Au niveau des formations sanitaires tertiaires, le résultat global de la PCI a donné un score de 75% soit un niveau moyen de satisfaction. Au niveau des formations sanitaires privées, l´évaluation globale était de 53% soit un niveau moyen de satisfaction. Enfin, celle des formations sanitaires publiques de premier niveau a donné un score de 45% soit un niveau très bas de satisfaction. Les mesures de PCI dans les formations sanitaires publiques et privées de Niamey demeurent un défi pour les autorités sanitaires. L´élaboration d´un plan de résolution des problèmes sera utile pour relever ce défi.

## Abstract (en)

Niger has been facing the coronavirus disease 2019 (COVID-19) pandemic since 19^th^ March 2020. In this article we report an assessment of infection prevention and control (IPC) practices at healthcare facilities in the city of Niamey in Niger and propose solutions. This assessment focused on the 12 themes contained in the World Health Organization IPC assessment framework for healthcare facilities. The assessment was conducted in 83 public and private healthcare facilities, which represent 60% of healthcare facilities in the city of Niamey. At the level of tertiary healthcare facilities, the overall IPC score was 75% which represents a moderate level of compliance with recommended IPC practices. At the level of private healthcare facilities, the overall score was 53%; also, a moderate level of performance. Finally, the overall IPC score was 45% at primary public healthcare facilities; which shows a very low level of adherence to IPC recommendations. IPC practices in public and private healthcare facilities in Niamey remain a challenge for healthcare authorities. Developing a tailored restorative plan would be helpful in meeting this challenge.

## Perspectives

Le Niger à l´instar des autres pays du monde fait face depuis le 19 mars 2020, à la pandémie de la maladie à coronavirus 2019 (COVID-19) [1,2]. Toutes les régions du pays sont atteintes par la pandémie. Selon le rapport de situation du Ministère de la Santé Publique à la date du 25 juin 2020 il a été enregistré un total de cas cumulés de 1059 répartis sur l´ensemble du territoire, dont la communauté urbaine de Niamey rapporte à elle seule 75,6% des cas (soit 801 cas) [3]. Au cours de cette pandémie, le personnel de santé, toutes catégories confondues, a payé un lourd tribut avec 184 cas sur 1059 cas confirmés soit 17,4% des cas confirmés [3]. Cet état de fait pourrait s´expliquer d´une part, par la non mise en œuvre effective des précautions standards en matière de prévention et contrôle des infections (PCI), et d´autre part par la nouveauté de la maladie dans le système de surveillance et de contrôle sanitaire. Face à ce problème, plusieurs actions ont été entreprises par la commission PCI au Niger avec l´appui financier de l´Organisation mondiale de la santé (OMS) et le Fonds des nations unies pour l´enfance (Unicef) tel que la présente évaluation des mesures PCI dans les formations sanitaires de Niamey, la fourniture des équipements de protection individuelle aux agents de santé, l´élaboration des modes opératoire standard (SOPs) sur l´enterrement digne et sécurisé et la PCI dans les formations sanitaires et dans la communauté. En outre, les formations des agents de santé de première ligne se sont poursuivies avec l´appui des autres partenaires à travers la formation de 500 agents de santé et 200 techniciens de surface des formations sanitaires publiques et privées de Niamey et 210 agents chargés du dépistage, de la détection précoce et de la prise en charge de la tuberculose et du VIH au niveau des sept autres régions hors de la communauté urbaine de Niamey. Aujourd´hui, grâce aux précautions de prévention usuelle envisagées dans toutes les formations sanitaires tant publiques que privées, les cas positifs chez le personnel de santé sont restés stationnaires. Cependant, il s´avère nécessaire de redoubler d´efforts pour minimiser les risques de propagation des infections dans ces établissements. C´est pourquoi l´objectif de cette étude était d´évaluer les mesures de PCI dans les structures sanitaires de la communauté urbaine de Niamey et d´élaborer un plan de résolution des problèmes.

Il s´agissait d´une analyse descriptive des 12 thématiques contenues dans la fiche Scorecard PCI de l´OMS [4,5] au niveau des formations sanitaires publiques et privées de de la communauté urbaine de Niamey au Niger. L´étude s´est déroulée du 23 juin au 03 juillet 2020. La population de l´étude a été constituée des formations sanitaires publiques et privées y compris les formations sanitaires tertiaires de la communauté urbaine de Niamey. Au total 140 structures sanitaires ont été identifiées dont sept formations sanitaires tertiaires (six hôpitaux et une maternité de référence), 62 formations sanitaires publiques de premier niveau (les centres de santé intégrés), et 71 formations sanitaires privées (les cliniques et cabinets médicaux). Les structures suivantes étaient éligibles à l'inclusion: toutes les formations sanitaires tertiaires (à cause de la forte fréquentation et le caractère référentiel), les services de santé des armées (à cause de la forte fréquentation), et les formations sanitaires avec au moins un agent de santé infecté au COVID-19. Ont été exclus: les structures où les responsables ont refusé de se prêter à l´interrogatoire, la fermeture de la structure sanitaire pour cause de COVID-19, et les structures ayant les procédures administratives trop serrées. La méthode d´échantillonnage non probabiliste de commodité a été utilisée pour sélectionner les formations sanitaires à évaluer. Au total 83 structures (sur les 140 structures existantes) ont été évaluées, avec 71% publiques (soit 59 structures dont sept formations sanitaires tertiaires et 52 centres de santé intégrés) et 30% privées (soit 25 cliniques et cabinets médicaux). Le Tableau 1 montre les structures qui ont fait l´objet de l´évaluation. Les techniques suivantes ont été utilisées pour collecter les données: (a) l´analyse documentaire (la vérification de la disponibilité des procédures normalisées, des directives, des termes de références, etc.); (b) l´entretien avec les responsables et agents des formations sanitaires visitées; et (c) l´observation de l´environnement des soins et des malades.

**Tableau 1:** les structures évaluées

Structures	District 1	District 2	District 3	District 4	District 5
**CSI**	Garde Nationale du Niger	Foulan Koira	Nouveau marché	Police (Camp Bano)	Karadjé
	Gendarmerie	Dar Es Salam	Banifandou	Gamkallé	Saguia
	Racasement	Ligue Islamique	Madina	Saga	Gawèye
	Yantala haut	Caisse Nationale de Sécurité Sociale Maourey	Abidjan	Talladjé	Lamordé 1
	Yantala bas	Agence Nigérienne pour le Bien Etre Famillial	Caisse Nationale de Sécurité Sociale Kalley	Wadata	Lamordé 2
	Magama	Boukoki	Prison civile	Aéroport 1	Banga bana
	Bobiel	Lazaret	Cité Fayçal	Aéroport 2	Zamagandey
	Institut de Formation aux Technologie de l'Information et de la Communication	Deyzeibon	République	Bassora	Néni goungou
	Goudel	Kombo	Boukoki IV	Rte Filingué	Yawaré
	Tondibiah	Nord Lazaret	Kongou Gorou	Saga gorou	Bougoum
	Matenité Koira Kano				
**FST**	Hôpital National de Niamey	Hôpital Général de Reference	Centre Hôpitalier Régional		Gawèye
	Hopital Turque		Maternité Issaka Gazoby		Hôpital National Amirou Boubacar Diallo
**Privées**	Afoua	Wangari	Rafani	Djoliba	Alhamdoulilah
	Akika	Lacouroussou	La cité	Maimouna	
	Martaba	Damergou	Magori	Rayane	
	Rayua	Rahamat	Kaocen	Fanta	
	Alissa	Tropical		Lahiya	
	Mali Béro	Déla Santé			
	As Salam				
	Alomar				
	Dr Sacko				

CSI, Centre de santé intégré; FST, Formations sanitaires tertiaires

La fiche d´évaluation de la PCI basée sur l´attribution des scores pour chaque thématique (version OMS) a été utilisée pour l´évaluation [4]. Afin d´obtenir l´adhésion des responsables des formations sanitaires aux objectifs de l´évaluation, une correspondance administrative émanant du Secrétariat Général du Ministère de la Santé Publique leur a été adressée, ainsi qu´au Président de la plateforme du Secteur privé de la santé. Ensuite, on a conduit une formation des évaluateurs. Cette formation a été centrée sur la fiche de collecte des données. Elle a également permis de mieux outiller les évaluateurs en termes de la COVID-19 et des mesures PCI. Au cours de cette session, un pré-test de l´outil a été organisé. L´outil de collecte des données a fait l´objet d´un pré-test au cours de la formation des évaluateurs dans cinq formations sanitaires qui n´ont pas été retenues pour l´évaluation. Ce qui a permis aux évaluateurs de se familiariser avec l´outil et discuter de certains aspects pratiques sur le terrain. Des corrections d´améliorations ont été apportées sur la fiche après ce pré-test. L´étude s´est déroulée du 23 juin au 03 juillet 2020 dans 83 formations sanitaires (Tableau 1). Les données recueillies ont été enregistrées dans un Dashboard (Excel). Deux aspects ont été pris en compte dans l´analyse des données: (1) Les données d´observations, d´analyses documentaires et d´entretiens rapportées sur la fiche; et (2) la détermination des scores pour chaque thématique. Sur la base du score global obtenu sur les 12 thématiques, chaque formation sanitaire a été classée dans l´un des trois niveaux de promotion et pratique de la PCI. Le Tableau 2 a permis de déterminer les niveaux de classification des formations sanitaires selon l´outil Scorecard PCI.

**Tableau 2:** différents niveaux de classification des FS selon l´outil Scorecard PCI

Score de performance		Signification	Suivi
Classification	<50%	La mise en œuvre des principales composantes de la PCI est déficiente. Une amélioration significative est requise	1 fois par jour
	50-79%	Certains aspects des principales composantes de la PCI sont en place, mais pas suffisamment mis en œuvre. D´autres améliorations sont nécessaires.	2 ou 3 fois par semaine
	>79%	La plupart des aspects des principales thématiques de la PCI sont mis en œuvre de manière appropriée. La formation devrait continuer à améliorer l´étendue et la qualité de la mise en œuvre et se concentrer sur l´élaboration de plans à long terme pour soutenir et promouvoir davantage les activités du programme de PCI existantes.	1 fois par semaine

Source: OMS

**La performance globale des formations sanitaires**: le taux de complétude était de 100% pour les formations sanitaires privées et les formations sanitaires tertiaires. Il était de 98% au niveau des centres de santé intégrés. Cela s´explique par le fait qu´au niveau de la prison civile, la formation sanitaire n´a pas pu être visitée à cause des contraintes liées aux procédures administratives. Au niveau des sept formations sanitaires tertiaires, le résultat global a donné un score de 75% avec un niveau de satisfaction moyen. On note également que 41% des thématiques (soit 5 sur 12) ont eu un score allant de 90% à 100% avec un niveau satisfaisant. Aucune thématique n´a reçu un score inférieur à 50%. Pour les structures sanitaires privées, l´évaluation globale des tous les 25 structures a donné un score de 53% avec un niveau moyen de satisfaction. Six thématiques sur douze (soit 50%) ont eu un score compris entre 50% et 79% avec un niveau moyen de satisfaction et six thématiques (soit 50%) ont eu un niveau insuffisant avec un score inférieur à 50%. Il s´agit de la formation du personnel, le triage des patients, l´existence d´une zone d´isolement, l´existence d´un point focal ou d´un comité d´hygiène, l´élimination des déchets, et l´exposition d´un agent au coronavirus. Aucune thématique n´a enregistré un niveau satisfaisant (c´est-à-dire plus de 79%). Au niveau des centres de santé intégrés, le niveau de performance globale était insuffisant (avec un score de 45%). Sur 51 formations sanitaires publiques évaluées, sur les 12 thématiques de la PCI ayant fait l´objet d´évaluation, 67% ont eu une performance insuffisante avec des scores allant de 8 à 46%. Quatre thématiques seulement sur les douze, ont connu une performance moyenne avec des scores allant de 54% à 72%. Aucune thématique n´a enregistré un niveau satisfaisant.

**L´existence d´un point focal ou d´un comité d´hygiène en place**: le résultat varie de 100% au niveau des formations sanitaires tertiaires, 44% au niveau des formations sanitaires privées, et 29% au niveau des centres de santé intégrés. Cette performance insuffisante au niveau des centres de santé intégrés et structures privées pourrait s´expliquer par l´absence des termes de références et leur connaissance par le point focal. Dans d´autres formations sanitaires, il s´agit de l´insuffisance de temps alloué au point focal suite aux chevauchements des activités.

**Le système de triage des patients:** il existe un système de tri des patients au niveau des formations sanitaires tertiaires avec une performance de 95%. Cependant le triage des patients reste défaillant dans certains centres de santé intégrés et structures privées où la performance est insuffisante avec des scores variant de 40% dans les formations sanitaires privées et 44% dans les centres de santé intégrés. Les insuffisances liées au tri des patients se résument à: (a) l´absence du contrôle de la température avec un thermoflash fonctionnel et la vérification des symptômes du COVID-19 à l´entrée des formations sanitaires. Au niveau des centres de santé intégrés, le score est 33% contre 67% au niveau des formations sanitaires privées; et (b) la non disponibilité et l´insuffisance dans l´utilisation des fiches des triages avec des scores variant de 32% dans les formations sanitaires privées à 43% dans les centres de santé intégrés.

**Identification d´une zone d´isolement**: la zone d´isolement est presque inexistante dans les formations sanitaires. La performance est moyenne au niveau des formations sanitaires tertiaires avec un score de 67%, tandis qu´au niveau des centres de santé intégrés et structures privées elle est insuffisante avec respectivement 8% et 39% de score. Cette situation pourrait s´expliquer par: (a) la non disponibilité d´une zone d´isolement et à l´écart des autres services dans certaines formations sanitaires: 18% dans les centres de santé intégrés et 40% dans les formations sanitaires privées; (b) la non disponibilité des toilettes dédiées dans la zone d´isolement ou présence d´un bassin de lit ou urinoir avec un variant de 43% dans les formations sanitaires tertiaires, 4% dans les centres de santé intégrés, et 44% dans les formations sanitaires privées; (c) l'insuffisance voire l´absence d´une station de lavage des mains et des fournitures (des équipements de protection individuelle, un lit, bassin ou urinoir, etc.), une zone pour mettre les équipements de protection individuelle et une zone pour enlever les équipements de protection individuelle (circuit bien respecté) dans chaque zone d´isolement. Sur ce point la performance est insuffisante dans toutes les formations sanitaires évaluées et varie de 4% dans les centres de santé intégrés à 32% dans les formations sanitaires privées.

**La pratique du lavage des mains ou les stations pour l´hygiène des mains**: la pratique du lavage des mains et ou la présence d´un dispositif prévu à cet effet existe dans toutes les formations sanitaires avec une performance moyenne pour des scores variant de 52% dans les formations sanitaires tertiaires, 54% dans les centres de santé intégrés, à 55% dans les formations sanitaires privées. Cependant certains critères ne sont pas suffisamment mis en œuvre ou sont presque inexistants. Les quelques formations sanitaires où le dispositif existe, on constate soit le manque de savon, l’eau ou parfois la non-fonctionnalité du dispositif. La technique de lavage des mains ou de friction des mains au gel hydro-alcoolique selon la technique OMS ne semble pas être suffisamment maitrisée par les agents. Il n’existe aucun poster sur les différentes techniques d’hygiène des mains au niveau de chaque station de lavage des mains.

**La disponibilité et usage des équipements de protection individuel**: la disponibilité et l´usage des équipements de protection individuelle est d´une performance moyenne au niveau des formations sanitaires privées et les formations sanitaires tertiaires de référence avec des scores respectifs de 56% et 57%. Par contre, elle est insuffisante au niveau des centres de santé intégrés. Dans l´ensemble, la disponibilité et l´usage des équipements de protection individuelle se limitent parfois à la seule disponibilité des gants et/ou des masques. Les blouses, lunettes, l´écran facial et les bottes ne sont pas disponibles ou sont insuffisantes dans certaines structures. Les posters sur les précautions standards du Ministère de la Santé en collaboration avec l´OMS sur comment mettre et enlever les équipements de protection individuelle sont inexistants et le personnel présente des insuffisances et n´est pas parfois en mesure de mettre et retirer les équipements de protection individuelle en suivant correctement l´ensemble des étapes.

**Existence d´un mécanisme de tri des déchets**: il existe un mécanisme de tri des déchets au niveau de toutes les structures sanitaires avec une performance moyenne. Le score varie de 76% pour les formations sanitaires tertiaires et les FS privées à 71% dans les centres de santé intégrés. Malgré cette performance moyenne, des insuffisances existent et sont liées à: (a) la non-disponibilité des poubelles étanches, couvertes et étiquetées (infectieux ou non-infectieux) et les affiches sur la gestion des déchets sont disponibles dans tous les points de service aux patients: 71% pour les formations sanitaires tertiaires, 68% pour les formations sanitaires privées et 55% pour les centres de santé intégrés; et (b) les déchets ne sont pas triés selon les types (infectieux, non infectieux, piquants ou tranchants): 72% pour les formations sanitaires privées et 49% pour les centres de santé intégrés.

**L´élimination des déchets**: l´élimination des déchets dans toutes les formations sanitaires évaluées constitue un défi. Les résultats obtenus font ressortir que beaucoup d´aspects ne sont pas suffisamment mis en œuvre et des améliorations pour y parvenir sont nécessaires. Pendant que la performance est moyenne dans les formations sanitaires tertiaires et les centres de santé intégrés avec respectivement un score de 52% et 55%, cette performance est insuffisante avec 47% de score dans les formations sanitaires privées. On peut dès lors constater dans certaines formations sanitaires que: (a) les déchets ne sont pas toujours brûlés sur place faute d´incinérateur ou d´un mécanisme prévu pour le transport dans un autre endroit approprié; (b) le port des équipements de protection individuelle appropriés (gants en latex ou en nitrile, gants de ménage, lunettes de protection, bottes en caoutchouc, tabliers et masques de protection) par le personnel lors de la manipulation des déchets n´est pas parfois systématique faute de disponibilité des équipements de protection individuelle; et (c) une fosse à placenta ou de déchet organique n´est pas disponible et serait dû probablement au contexte socioculturel qui exige aux individus de disposer de leurs organes. Cependant cette fosse doit exister pour l´enfouissement des parties organiques pathologiques.

**La formation du personnel:** la formation constitue un élément essentiel dans la mise en œuvre des précautions standards en matière de PCI en milieu de soins. Les résultats de l´évaluation font ressortir une performance moyenne pour les formations sanitaires tertiaires et centres de santé intégrés avec un score respectif de 57% et 72%, cependant les formations sanitaires privées enregistrent une performance insuffisante avec un score de 39%.Les résultats performants observés au niveau des formations sanitaires tertiaires et centres de santé intégrés sont à mettre à l´actif de la formation sur la PCI reçue par 500 agents de santé toutes catégories confondues et 200 techniciens de surface et hygiénistes. Par cette formation, au niveau des formations sanitaires tertiaires, 71% ont reçu une formation minimale sur les précautions standards, les précautions additionnelles (périodes pratiques et théoriques) et un accent sur COVID-19 dans les derniers 6 mois. Au niveau des centres de santé intégrés, 90% disposent au moins d´un agent formé sur les précautions standards (pratiques et théoriques) avec un accent sur le COVID-19 dans les derniers 6 mois. Au niveau des formations sanitaires privées le score est de 52%. Cependant il existe des insuffisances telles que le manque de transfert des connaissances, l´insuffisance de la supervision formative et l´absence de la documentation avec description des formations reçues par le personnel.

**Alerte des cas suspects intra-hospitalier (niveau des formations sanitaires)**: le système d´alerte au niveau des formations sanitaires tertiaires est satisfaisant avec un score de 90%. Cependant ce système présente des insuffisances en ce qui concerne la visite des hospitalisés pour la détection des cas suspects et leur déplacement dans la zone d´isolement. Par ailleurs le système d´alerte est d´une performance moyenne au niveau des formations sanitaires privées avec un score de 63% et insuffisante dans les centres de santé intégrés qui constituent le premier niveau des soins où le score est de 41%.

**La stérilisation**: la performance est moyenne au niveau des formations sanitaires tertiaires et formations sanitaires privées avec respectivement un score de 76% et 67%. Une performance insuffisante avec un score de 41% est observée dans les centres de santé intégrés. Du reste il ressort que cette situation présente certaines insuffisances notamment la non-disponibilité des SOP dans certaines formations sanitaires sur la manière d´effectuer la stérilisation du matériel/ équipement et la formation du personnel en charge.

**Le bio-nettoyage de l´environnement du patient**: le bio-nettoyage de l´environnement du patient a une performance satisfaisante avec un score de 100% au niveau des formations sanitaires tertiaires. Elle va d´une performance moyenne avec un score de 67% dans les formations sanitaires privées, à une performance insuffisante avec un score de 46% dans les centres de santé intégrés. La disponibilité permanente d´une source d´approvisionnement en eau potable et de stockage pour pallier aux arrêts momentanés de la fourniture d´eau, est à un score allant de 49% (insuffisant) dans les centres de santé intégrés, 71% (moyenne) dans les formations sanitaires tertiaires est de 84% (satisfaisante) dans les formations sanitaires privées. Des insuffisances constatées se résument à la non-disponibilité des SOP sur le nettoyage ou désinfection, lorsqu´il y a des liquides corporels ou déversements de sang et le nettoyage et décontamination du matériel réutilisable. La formation du personnel reste également un défi dans ce domaine.

**Exposition d´un agent de santé au coronavirus**: l´exposition d´un agent de santé semble bien être sous contrôle au niveau des hôpitaux avec un score de 90% pour une performance satisfaisante. Au contraire, il existe des insuffisances dans les centres de santé intégrés (33%) et formations privées (45%) avec une performance insuffisante. Ces insuffisances sont caractérisées par le manque de protocole d´évaluation et prise en charge en cas d´exposition (incluant un registre, les outils d´évaluation, communication, etc.). La prise en charge du personnel de santé exposé qui n´est pas clairement définie et parfois non assurée. Devant ces insuffisances constatées, un plan de résolution des problèmes élaboré et mis en œuvre avec un suivi régulier pourrait réduire les insuffisances à la limite des objectifs fixés. L´analyse des données fait ressortir des insuffisances au niveau des 12 thématiques et pour toutes les catégories de formations sanitaires. Pour une meilleure amélioration de la mise en œuvre des mesures PCI dans ces formations sanitaires, la mise en œuvre du plan de résolution des problèmes doit non seulement s´articuler autour de la formation du personnel de santé, mais aussi dans un processus de mise en place des équipements de protection individuels, des intrants, et assurer des investissements pour la disponibilité d´un système d´approvisionnement en eau potable et d´assainissement solide. Même si les éléments ci haut cités sont assurés au niveau de toutes les formations sanitaires il n´en demeure moins que le système à tous les niveaux doit aussi garantir un mécanisme adéquat en ce qui concerne le tri des malades et aussi le tri et le traitement des déchets, d´où la nécessité de disposer des infrastructures à cet effet (local pour l´isolement des malades, des poubelles pour le tri des déchets et des incinérateurs pour la destruction des déchets). Cela suppose une appropriation du plan de résolution des problèmes par les responsables pour la mise en œuvre et le suivi au niveau des districts sanitaires de rattachement des formations sanitaires évaluées.

## Conclusion

Les mesures de prévention et de contrôle des infections dans les formations sanitaires tant publiques que privées demeurent un défi pour les autorités sanitaires. La mise en œuvre des précautions standards vise non seulement à protéger les prestataires eux-mêmes, mais aussi les utilisateurs des services de santé (patients) et la communauté. L´évaluation des 83 (60%) formations sanitaires de la communauté urbaine de Niamey a permis de relever ces insuffisances à la mise en œuvre des précautions standards pour la PCI en milieu de soins. Ces insuffisances sont identifiées au niveau de toutes les thématiques de la PCI selon le Scorecard PCI de l´OMS [4].

## Remerciements

Nous remercions l'OMS d'avoir financé l'étude rapportée dans cet article.

## References

[1] Rosenthal PJ, Breman JG, Djimde AA, John CC, Kamya MR, Leke RGF (2020). COVID-19: shining the light on Africa. Am J Trop Med.

[2] Cabore JW, Karamagi HC, Kipruto H, Asamani JA, Droti B, Seydi ABW (2020). The potential effects of widespread community transmission of SARS-CoV-2 infection in the World Health Organization African Region: a predictive model. BMJ Glob Health.

[3] Ministère de la Santé Publique (2020). Secrétariat général: comité technique de gestion de la réponse à la pandémie du Coronavirus (COVID-19), Niger, pandémie coronavirus (COVID-19): rapport de situation N^o^81 du 25 juin 2020. Niamey, Niger.

[4] World Health Organization Infection prevention and control assessment framework at the facility level.

[5] World Health Organization Guidelines on core components of infection prevention and control programmes at the national and acute health care facility level.

